# Full genome–based evolutionary analyses of FMD virus serotype A including field outbreak strains isolated from India during the period 2008–22

**DOI:** 10.1093/ve/veaf097

**Published:** 2025-12-18

**Authors:** Jajati Keshari Mohapatra, Biswajit Das, Saravanan Subramaniam, Shyam Singh Dahiya, Manoranjan Rout, Bikash Ranjan Prusty, Rabindra Prasad Singh

**Affiliations:** ICAR-National Institute on Foot and Mouth Disease, Indian Council of Agricultural Research, International Centre for FMD, Arugul, Bhubaneswar 752050, Khordha, Odisha, India; Department of Biological Chemistry and Molecular Pharmacology, Blavatnik Institute, Harvard Medical School, Boston, MA 02115, United States; ICAR-National Institute on Foot and Mouth Disease, Indian Council of Agricultural Research, International Centre for FMD, Arugul, Bhubaneswar 752050, Khordha, Odisha, India; ICAR-National Institute on Foot and Mouth Disease, Indian Council of Agricultural Research, International Centre for FMD, Arugul, Bhubaneswar 752050, Khordha, Odisha, India; ICAR-National Institute on Foot and Mouth Disease, Indian Council of Agricultural Research, International Centre for FMD, Arugul, Bhubaneswar 752050, Khordha, Odisha, India; Nanaji Deshmukh Veterinary Science University (N.D.V.S.U.), Adhartal, Jabalpur, Madhya Pradesh Pin- 482004, India; ICAR-National Institute on Foot and Mouth Disease, Indian Council of Agricultural Research, International Centre for FMD, Arugul, Bhubaneswar 752050, Khordha, Odisha, India

**Keywords:** foot-and mouth disease, serotype a, A/ASIA/G-18/2019, comparative genomics

## Abstract

Comparative complete genome analyses were conducted on 173 field outbreak strains of foot-and-mouth disease virus (FMDV) serotype A, collected from various regions worldwide, including strains that have circulated in India in recent years. Phylogenetic analyses revealed that the majority of isolates included in this study belonged to the Asia (*n* = 108), followed by EURO-SA (*n* = 41) and Africa (*n* = 24) topotypes. The mean rate of evolutionary change in FMDV serotype A was estimated to be 2.369 × 10^−3^ substitutions/site/year for the Open Reading Frame (ORF). Faster substitution rates in the Asia topotype suggests heightened selective pressures, likely driven by pre-existing host immune responses due to prior infections or vaccination. The periodic emergence and subsequent dominance of notable genotypes or lineages within the Asia topotype such as genotype 18 (ASIA/VII), genotype 20 (Sea-97), and genotype 26 (Iran-05) underscore the ongoing diversification, adaptation, and selection of the virus in the field across Asia. Monophyletic clustering within the Asia and Africa topotypes suggests region-specific evolutionary trajectories, while the diversity observed within EURO-SA indicates an older and more genetically varied lineage pattern. The presence of amino acid insertions and deletions in some of the isolates points to potential hotspots for genetic change, particularly in regions such as L, VP1, and 3A, reflecting high genetic volatility. Positive selection across the protein-coding regions excluding VP4 and 2A highlights the virus’s adaptive potential, likely contributing to immune evasion, host adaptation, and enhanced fitness for replication and transmission. Evidence of recombination events, particularly in five isolates with spatio-temporal overlaps, indicates dynamic viral evolution potentially favourable for emergence of new variants. These findings are crucial for understanding foot-and-mouth disease (FMD) epidemiology and may have implications for global FMD control strategies.

## Introduction

Foot and mouth disease virus (FMDV) is a positive-strand RNA virus of the genus *Aphthovirus* within the family *Picornaviridae* that infects a wide range of domestic and wild cloven-hoofed animals. Among the three prevalent serotypes (O, A, and Asia 1) in India and neighbouring countries in South Asia, serotype A is considered to be genetically and antigenically the most divergent one (Subramaniam et al. 2022). A vaccination-based FMD control programme has been in operation since 2003–04 in India. An increase in the number of FMD outbreaks in recent years (2021–23) due to serotype A, which used to be associated with sporadic occurrence, has been observed (ICAR-NIFMD, Annual Report 2022 and 2023, https://www.nifmd.res.in/Institute_publication.php). Globally, serotype A could be divided into 26 genotypes (1–26) based on the complete VP1 gene sequence diversity with >15% nucleotide divergence among the genotypes (Mohapatra et al. 2011a). All genotypes are classified within one of three continental topotypes, which represent geographically clustered genetic lineages. Four genotypes (2, 10, 16, and 18) of FMDV serotype A have been identified in India to date. Genotypes 2 and 10 have not been detected since 1990, and genotype 16 has not been reported after 2001. Co-circulation of genotype 16 and 18 was documented during 1990–2001, followed by the exclusive presence of genotype 18 (earlier designated as genotype VII).

The genotype 18 has been in circulation for a very long time and has had the highest epidemiological impact, among all the lineages/genotypes that have circulated ever in India. Within genotype 18, the VP3^59^-deletion group, having an amino acid deletion at the 59th position of VP3, appeared during the later part of 2002 (Jangra et al. 2005). Since then, both the VP3^59^-deletion and nondeletion groups of viruses within the genotype 18 are responsible for all the serotype A outbreaks reported in the country. The emergence of a novel genetic group within genotype 18 was documented in 2019, which clustered distinctly from both the VP3^59^-deletion and nondeletion lineages in the VP1-based maximum likelihood phylogeny. Examination of the VP3 region revealed the presence of the 59th amino acid, and this group was designated as the ‘A/ASIA/G-18/2019’ lineage (Mohapatra et al. 2023). The novel lineage, first reported in 2019, appears to have established a strong foothold in India and has also been reported in Bangladesh. Most importantly, the majority of strains belonging to this novel lineage exhibit a poor antigenic match with the Indian vaccine strain in use, A/IND40/2000 (Mohapatra et al. 2023).

Genetic characterization of field outbreak strains, particularly comprehensive complete genome sequence analyses on a continuous and real-time basis, is essential to understand their origin, evolution, fitness, movement, transboundary spread, and global distribution with high resolution under natural conditions. Conventionally, genetic analyses of FMDV serotype A strains in India have primarily focused on deciphering the structural protein-coding region or specific nonstructural protein-coding regions/untranslated region (UTRs) of interest. To date, only two reports on the complete genome sequence analyses are available: one involving field strains that circulated till 2008 (Subramaniam et al. 2011) and another on the vaccine strains (Mohapatra et al. 2011b). In this study, complete genome sequences of recent serotype A Indian FMD viruses, collected from field outbreaks between 2008 and 2022, including the novel lineage A/ASIA/G-18/2019, were determined and analysed alongside publicly available sequences to gain insights into their molecular epidemiology and evolutionary trends.

## Materials and method

### Isolates

Four FMDV serotype A-positive samples belonging to the novel lineage ‘A/ASIA/G-18/2019’, collected from field outbreaks during 2019–22 and 18 serotype A isolates (year of origin 1988 and 2008–13) obtained from the repository of ICAR-National Institute on FMD were sequenced (Supplementary Table S1). The passage level of the isolates in BHK-21 cell culture was kept minimal at about three to five passages before they were subjected to nucleotide sequencing.

### Nucleotide sequencing

Full genome sequences were determined for 18 archived isolates by capillary Sanger sequencing. The details of PCR and sequencing primers used and thermal conditions are essentially as described earlier by us (Mohapatra et al. 2011b). The complete genome was amplified in seven overlapping fragments using *Pfu* DNA polymerase (Fermentas), which possesses proofreading activity. Overlapping sequence reads were generated to reduce the probability of sequencing errors. Cycle sequencing reactions of gel-purified PCR products were carried out using the BigdyeV3.1 terminator kit on the ABI 3130 genetic analyser (Applied Biosystems). Sequence contigs were processed manually from the overlapping fragments generated by forward and reverse primers.

Full genome sequences for four isolates of the novel lineage were determined by next-generation sequencing. The RNA obtained from virus-infected BHK-21 cell supernatants was sequenced using an Illumina Novaseq6000 platform (Illumina, USA). Library preparation was performed following the Illumina TruSe*q RNA* library protocol as outlined in the TruSeq*RNA Sample Preparation Guide* (Illumina). The Illumina paired-end (2 × 150 bp) raw reads were quality-checked using FastQC. High-quality reads were aligned to the reference sequence using BWA-MEM (version 0.7.17), and consensus sequences were extracted using SAMtoolsmpileup.

### Phylogenetic analysis

Complete coding region sequences of 22 isolates generated in this study were aligned with 151 FMDV serotype A complete genome sequences available in the GenBank (Supplementary File S2). All publicly available FMDV serotype A sequences, accessed as of December 2024, were included in this study irrespective of their geographic origin, year of collection, or other associated metadata. This inclusive approach was adopted to ensure maximum representation of the prevailing global sequence diversity and to avoid potential selection bias, thereby enhancing the robustness and comprehensiveness of the analysis. The alignment was performed using MAFFT version 7 with the --auto option to ensure optimal alignment quality and accuracy (Katoh et al. 2019). Following alignment, the best-fit nucleotide substitution model was identified using IQ-TREE (Kalyaanamoorthy et al. 2017). The mean and pairwise divergence at the complete genome and various protein-coding regions were then computed. To assess the evolutionary relationships among FMDV serotype A viruses, phylogenetic trees were inferred by the maximum likelihood (ML) method based on the nucleotide alignment of the complete Open Reading Frame (ORF) and VP1 sequences, separately. The ML phylogeny was produced under the GTR evolution model with rate variation following a gamma distribution and robustness of the tree topology was assessed by bootstrap analysis with 1000 iterations using MEGA 11 software v. 11 (Tamura et al. 2021).

### Evolutionary and time scale analysis

The reconstructed ML nucleotide trees were utilized in TempEst (Rambaut et al. 2016) to produce linear regression plots of the years of sampling versus root-to-tip distance in order to examine the temporal signal. Using time-stamped sequence data with a relaxed and an uncorrelated lognormal clock under the Bayesian Markov chain Monte Carlo (MCMC) method, BEAST version 1.10.4 (Suchard et al. 2018) was used to estimate the rates of evolutionary change (nucleotide substitutions per site per year) and time of circulation of the MRCA (years). An asymmetrical state transition model was used to predict the discrete-state ancestral reconstruction of viral sampling locations (Lemey et al. 2009). The FigTree application v.1.4.0 was used to visualize and colour trees. The statistical uncertainty in the parameter estimates across the sampled trees was reflected in the 95% highest probability density (HPD) intervals.

### Selection pressure analysis

Two likelihood approaches, the single likelihood ancestor counting (SLAC) method and the fixed effects likelihood (FEL) method, and a Bayesian strategy called FUBAR were employed to determine the positive selection pressure at certain codon sites. In general, posterior probability > 0.9 for FUBAR and *P* < .1 for SLAC strongly imply positive selection. The Mixed Effects Model of Evolution (MEME) was used to identify the codon sites that were the subject of episodic diversifying selection. Strong evidence of selection was accepted at significance levels (*P* < .05). All the analyses were carried out using the online Datamonkey webserver (Weaver et al. 2018).

### Recombination analysis

The complete ORF sequence alignment was examined for any signs of recombination using the RDP5 software v. 5.30 (Martin et al. 2020). The analysis was carried out using various methods available and their default parameters. Recombination events were only deemed proven if they were identified by all the seven applied algorithms (RDP, Geneconv, BootScan, MaxChi, Chimaera, SiScan, and 3Seq) with default values.

## Results

### Phylogenetic relationships

**Figure 1:**
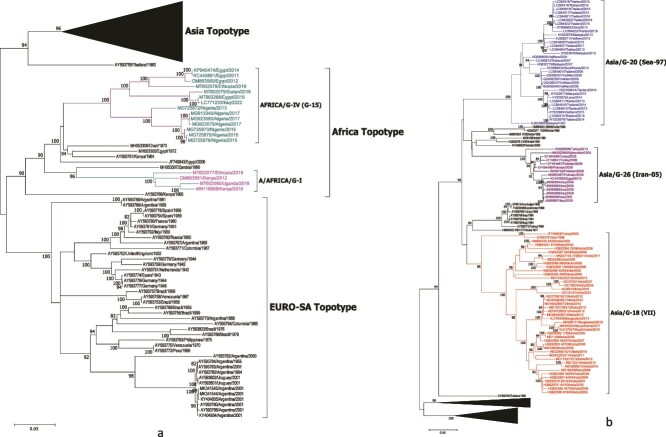
Phylogenetic tree reconstructed from 173 complete FMDV serotype A genomes sampled from different geographical regions. The tree was constructed using the maximum likelihood method with 1,000 bootstrap resampling iterations. Only bootstrap values greater than 80% are shown. Two genotypes under the Africa topotype (a) and three genotypes under the Asia topotype (b), which are currently in active circulation, are indicated using different colours.

For phylogenetic analysis, a total of 173 FMDV serotype A whole-genome sequences from 39 countries, representing four continents, comprising 151 sequences retrieved from the GenBank and 22 sequences deciphered in this study, were utilized. The VP1 region-based ML phylogenetic analyses indicated that the serotype A isolates grouped within three continental topotypes: Asia, Africa, and EURO-SA, as identified earlier (Mohapatra et al. 2011a). The majority of sequences (*n* = 108) included belonged to the Asia topotype, followed by EURO-SA (*n* = 41) and Africa (*n* = 24) (Fig. 1 and Supplementary Table S3). Nevertheless, we do not claim that the number of sequences included in this study reflects the actual number of outbreaks in a given region. Certain parts of the world may be underrepresented due to limited surveillance systems and lack of genetic data, which has consistently posed a challenge to phylogeographic analyses. Within the Asia topotype, the isolates were placed in seven genotypes, with the maximum number of isolates clustering within genotype 18 (ASIA/VII), which has been in circulation for about three decades now in the Indian sub-continent, genotype 20 (Sea-97) reported in Southeast Asia, and genotype 26 (Iran-05) prominent in Pakistan and the Middle East. All 22 Indian isolates sequenced in this study were grouped within genotype 18 (ASIA/VII), with 14 isolates in the VP3^59^-deletion group, 4 isolates in the nondeletion group, and 4 most recent isolates in the novel ‘A/ASIA/G-18/2019’ lineage. Within the EURO-SA topotype, the sequences clustered in eight genotypes; all of them seem to be extinct and were reported only prior to 2001. Although a EURO-SA lineage virus was recently reported in Egypt in 2022 (Hagag et al. 2023), it could not be included in the analysis due to the unavailability of its complete genome sequence in the public domain. Among the recent isolates for which full genome sequences were available, the majority (*n* = 14) of African topotype viruses clustered within A/AFRICA/G-IV and were collected in Algeria, Nigeria, Sudan, Iraq, Egypt, and Ethiopia during 2011–22. Four isolates sampled from Uganda, Kenya, and Ethiopia during 2012–19 grouped within A/AFRICA/G-I, and one isolate sampled in Egypt in 2006 clustered within A/AFRICA/G-VII. The genetic groups A/AFRICA/G-IV and A/AFRICA/G-VII were identified earlier in genotype classification studies and belong to genotypes 15 and 17, respectively (Mohapatra et al. 2011a).

### Evolutionary rate, time to most recent common ancestor (tMRCA), and population size changes

The root-to-tip regression analysis showed a correlation coefficient range of 0.221–0.827 and a coefficient of determination (R2) of 0.049–0.684 for the ORF and different coding regions (Table 1). The capsid region genes exhibited higher temporal signal in general, with the VP1 region displaying the maximum, followed by VP3 and VP2. Among the nonstructural coding regions, only the L region showed a significant temporal signal. The mean rate of evolutionary change in FMDV serotype A was estimated to be 2.369 × 10^−3^ subs/site/year (95% HPDs of 1.068–3.128 × 10^−3^ subs/site/year) for the ORF. The substitution rate of each coding region was compared, and the estimates descended in the following order: VP4, VP1, L, VP3, and VP2 (Table 1). Overall, the mean rate of evolutionary changes of different genomic regions varied from 2.996 × 10^−3^ to 3.427 × 10^−3^. The mean evolutionary rate of the capsid region of serotype A was estimated to be 3.086 × 10^−3^ nucleotide substitutions per site per year (95% HPDs of 2.332–3.657 × 10^−3^). The Asian topotype of serotype A displayed a considerably faster substitution rate (3.677 × 10^−3^ s/s/y; 95% HPD 2.993–4.398 × 10^−3^ s/s/y) than Africa (7.359 × 10^−4^ s/s/y; 95% HPD 4.017 x 10^−4^ to 1.093 × 10^−3^ s/s/y) and EURO-SA topotypes (4.638 × 10^−5^ s/s/y; 95% HPD 2.625 x 10^−6^—1.063 × 10^−4^ s/s/y).

**Table 1:** Rate of evolution and tMRCA predicted for different genomic regions of FMDV serotype A isolates

Genomic region	Rate of evolution	tMRCA	Correlation	R squared
L	3.238 ⨯ 10^−3^ (2.584 ⨯ 10^−3^—3.866 ⨯ 10^−3^)	1879.6 (1829.7–1914.3)	0.710	0.504
VP4	3.427 ⨯ 10^−3^ (2.441 ⨯ 10^−3^– 4.324 ⨯ 10^−3^)	1894.0 (1851.7–1925.9)	0.752	0.564
VP2	2.996 ⨯ 10^−3^ (2.408 ⨯ 10^−3^– 3.631 ⨯ 10^−3^)	1882.1 (1845.8–1911.1)	0.763	0.582
VP3	3.09 ⨯ 10^−3^ (2.361 ⨯ 10^−3^– 3.621 ⨯ 10^−3^)	1890.1 (1851.2–1919.0)	0.823	0.677
VP1	3.414 ⨯ 10^−3^ (2.842 ⨯ 10^−3^– 4.011 ⨯ 10^−3^)	1893.4 (1862.6–1919.4)	0.827	0.684

**Figure 2:**
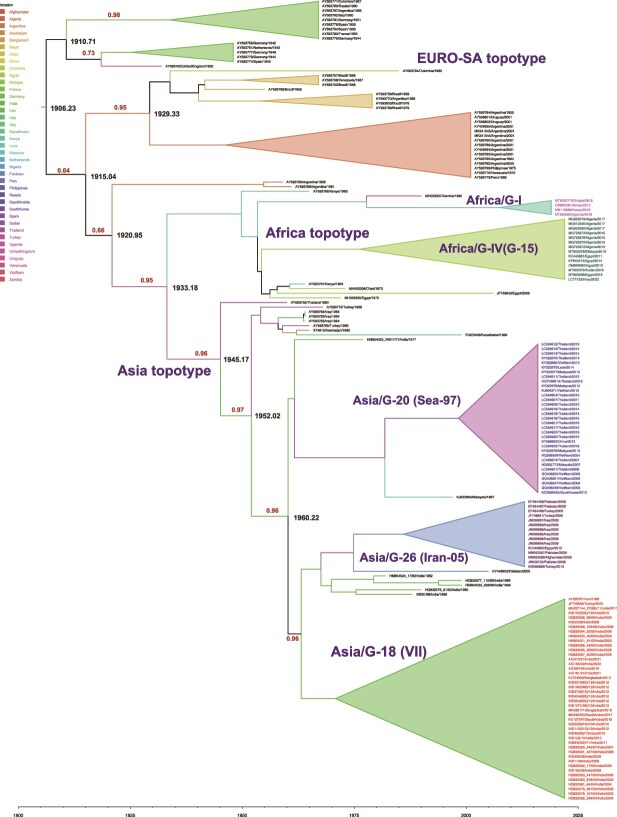
Maximum Clade Credibility (MCC) tree of the 173 FMDV serotypes A dataset was reconstructed under the assumption of a relaxed molecular clock. The analysis was performed on complete ORF. Posterior probability values for the major nodes are shown above the branches. Taxa are color-coded according to their geographical origin. The divergence time (time to the most recent common ancestor) is provided for each major node.

Based on the ORF, the tMRCA of FMDV serotype A isolates could be traced back to 1872.4 CE, with a 95% HPD interval of 1781.9–1914.2 CE. The analyses based on the capsid also yielded a similar tMRCA estimate of 1881.3 CE (95% HPD 1837.1–1915.8 CE). The estimated tMRCA derived from individual gene sequences spanned the period from 1879 to 1894, with L providing the lower estimate while VP4 providing the upper estimate (Table 1). The EURO-SA topotype is constituted by much more diverse samples and distinct genotypes with tMRCA estimated at 1873.2 CE with 95% HPD of 1791.3–1913.2 CE) (Fig. 2). In contrast, Asia and Africa topotypes maintained monophyletic clustering. The most recent common ancestor of the Asia topotype was located at ~1914.6 CE (95% credibility interval: 1803.5–1949.9 CE) and of the Africa topotype strains was located at 1914.4 CE (95% credibility interval: 1807.1–1949.9 CE). The root state probabilities estimated based on ORF ranged from 0.0001 to 0.3637 with Argentina and Germany receiving almost equal probability value of 0.3637 and 0.3479, respectively. Turkey and Uganda received a root state probability of 0.2471 and 0.2158, respectively for the common ancestor from which the Asia and Africa topotypes diverged.

**Figure 3:**
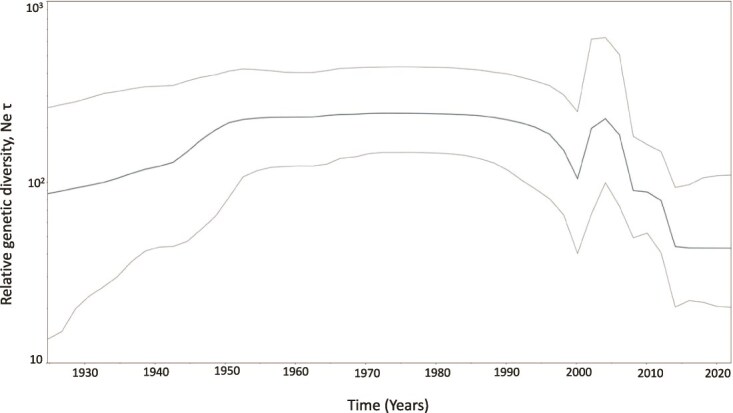
Demographic history of FMDV Serotype A isolates. Population growth was inferred via a Bayesian Skyline coalescent tree based on the capsid coding region. Fluctuations in effective population size (y-axis) over time (calendar years, x-axis) are presented. The thick central line indicates the median effective population size, while the thin lines above and below represent the 95% HPDs.

The change in epidemic history and evolutionary dynamics of FMDV serotype A over time was estimated based on the capsid coding region using the Bayesian Skyline Plot (BSP) model, and uncertainty in the estimated parameters was depicted using 95% Highest Probability Density (HPD) intervals (Fig. 3). The effective number of infections went through a period of smooth and steady increase from 1950 continuously through 1995, followed by a significant drop in the rate of spread in 2000. Noticeably, a sharp but transient increase in relative genetic diversity was observed from 2004 to 2006. This sharp peak was followed by a decline in the effective population, indicating purifying selection against the previously dominating strains.

### Selection pressure

In order to determine the influence of selection pressure on the evolution of serotype A, the ω value was calculated as the ratio of nonsynonymous (dN) to synonymous substitutions (dS). The global ω value of the serotype A dataset at the ORF (ω = 0.0847) was below 1.0, suggesting a lack of detectable positive selection pressure operational on the genome as a whole. The VP1 coding region had the highest ω value, followed by 3A, 3B, and L. Evidence of positive selection was found in all the individual protein-coding regions except VP4 and 2A. The maximum number of positively selected codon sites was found in the VP1 region (Table 2). The majority of the codon positions, ranging from 68% in 3B to 94% in VP3, were found under negative selection. The MEME approach predicted episodic selection at 82 codon sites in the coding region. 3D (*n* = 21) had the highest number of codon sites subject to episodic selection, followed by VP1 (*n* = 16). Out of the seven codon positions in the capsid coding region identified to be under positive selection by site-specific models, three positions (VP1–201 and VP3–59 and 175) are at predicted antigenically critical positions. Additionally, seven sites (VP1–141, 143, 150, 170, 173 and 194, and VP2–79) experiencing episodic selection are also found at antigenically critical positions, mostly within the surface-exposed loops.

**Table 2:** Codon positions found to be under positive and episodic selection in different genomic regions of FMDV serotype A. Numbering of codon position as per the Indian vaccine strain A IND40/2000 sequence. Proportions of sites under negative selection are shown within brackets

Genomic region	dN/dS	SLAC	FUBAR	MEME (episodic selection)
L	0.11	34, 47 (79.4%)	34, 47 (88.7%)	24, 25, 26, 34, 47, 80, 118, 154, 170, 188
VP4	0.02	Nil	Nil	Nil
VP2	0.07	134 (88.5%)	Nil (93.1%)	55, 65, 74,79,88, 96, 172, 173, 193, 195
VP3	0.07	175 (89.1%)	59, 175 (94.1%)	17, 65, 71, 80, 105, 175
VP1	0.16	43, 134, 171, 201 (76.5%)	171, 201 (84.5%)	22, 45, 60, 61, 96, 99, 110, 133, 141, 143, 150, 170, 173, 194, 196, 201
2A	0.03	Nil	Nil	Nil
2B	0.04	34, 121(71.0%)	34, 121 (84.4%)	34, 128
2C	0.05	83 (79.9%)	83 (88.7%)	4, 7, 12, 36, 55, 242, 259, 282, 257
3A	0.15	134, 146 (73.2%)	149 (78.4%)	128, 138, 139, 149
3B	0.15	4 (65.3%)	4 (68.1%)	4
3C	0.04	Nil (79.8%)	Nil (92.0%)	65, 106
3D	0.06	262 (74.5%)	262, 469 (84.9%)	55, 72, 76, 87, 123, 145, 148, 149, 205, 221, 262, 290, 291, 329, 355, 358, 379, 407, 437, 447 449

### Recombination

**Figure 4:**
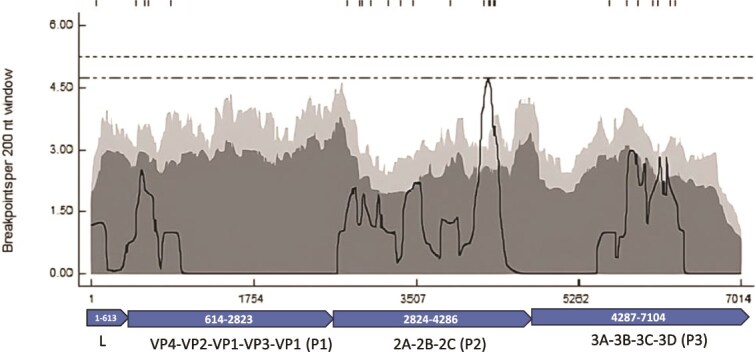
Recombination distribution plot. Small vertical lines at the top of the graph indicate each identifiable unique breakpoint site. The 99% and 95% confidence thresholds for globally significant breakpoint clusters are represented by the upper and lower dashed lines, respectively. The dark grey and light grey area are 95% and 99% confidence intervals, respectively, for local breakpoint clusters. The panel below the figure indicates the positions of the open reading frames of the FMDV genome (L, P1, P2 and P3).

In this study, 13 putative recombination events were identified through the analysis of 173 FMDV strains. (Table 3). These recombination signals could be detected within the same genetic group (within the VP3^59−^deletion group), between two different genetic groups of the same genotype (between the VP3^59−^deletion and nondeletion groups of genotype 18) and also between two different genotypes. Only five recombination events showed overlap in both time and space. To determine recombination hot and cold spots, a recombination breakpoint distribution plot was generated using a 200 nt window and 1000 permutations. No global hot-spot regions were observed at the 95% and 99% confidence thresholds (Fig. 4 and Supplementary Fig. 4). The detectable recombination breakpoint positions were mostly distributed over the non-structural protein (NSP) coding region.

**Table 3:** Putative recombination events in FMDV serotype A detected by all six default methods (RDP, GENECONV, BootScan, MaxChi, chimaera, SiScan, and 3SEQ (*P* < .01). The recombination events with probable spatial and temporal overlap are shown in boldface

Recombinant	Major parent	Minor parent	Beginning break point	Ending break point	Recombination score
**HQ832592_17/09/India/2009**	**HM854025_40/00/India/2000**	**HQ832590_245/07/India/2007**	ND	3 183	0.72
**HQ832590_245/07_India/2007**	**HQ832591_437/08_India/2008**	**HQ832579_281/03_India/2003**	5 877	UD	0.75
AY593776/Germany/1944	AY593759/Germany/1942	AY593792/Italy/1950	2 753	6 231	0.76
LC564908/Thailand/2012	LC564917/Thailand/2015	LC564904/Thailand/2010	4 334	5 871	0.75
LC564907/Thailand/2011	KJ933864/Malaysia/1997	LC564916/Thailand/2014	125	3 454	0.68
HQ832577_110/99_India/1999	HM854023_258/99_India/1999	HQ832586_43/06_India/2006	6 290	6 987	0.53
**IND84(202)/11_India/2011**	**HQ832591_437/08_India/2008**	**IND264(605)/12_India/2012**	6 043	6 791	0.72
AY593751_Netherlands/1942	Unknown	AY593774_Spain/1943	4 664	6 967	0.59
MN227144_27(68)/11/India/2011	Unknown	IND53/08/India/2008	307	2 743	0.74
HQ832587_50/06/India/2006	Unknown	HM854025_40/00/India/2000	473	2 922	0.58
AIC69/19/India/2019	HQ832586_43/06_India/2006	AIC195/22/India/2022	UD	2 833	0.68
**IND84(202)/11/India/2011**	**HQ832591_437/08/India/2008**	**IND264(605)/12/India/2012**	6 043	6 791	0.71
**HQ832586_43/06_India/2006**	**KY446902/Pakistan/2005**	**HM854025_40/00_India/2000**	241	2 919	0.56

### Genome variations in the global dataset

The overall nucleotide identity among all 173 analysed genome sequences was 84.6%. The ORF exhibited a maximum nucleotide divergence of 15.5%, with a mean variation of 12%. Among the regions coding for different genes, the VP1 region showed a mean divergence of 18%, followed by the L region, which exhibited 16% divergence (Table 4). The region with the least divergence was 2B, with a mean nucleotide variation of 9%. The highest nucleotide divergence was observed in the L region, at 26.3% translating into 21.1% divergence at the amino acid level, while the highest amino acid variation was observed in the 3A region, at 23.7%. Both these genetic segments have been shown to be dispensable for virus replication either in the natural or experimental setup.

**Table 4:** Nucleotide and amino acid variation in different genomic regions of FMDV serotype A

Genomic region	nt		aa	
	Mean divergence	Max divergence	Mean divergence	Max divergence
L	16.0	26.3	10.0	21.1
VP4	13.0	20.4	2.0	8.2
VP2	15.0	21.7	7.0	14.2
VP3	14.0	20.9	6.0	11.8
VP1	18.0	25.7	13.0	19.7
2A	9.0	25.0	1.0	18.6
2B	7.0	12.3	2.0	7.1
2C	9.0	18.3	3.0	9.1
3A	11.0	24.3	9.0	23.7
3B	8.0	23.0	5.0	22.5
3C	9.0	15.2	2.0	8.9
3D	9.0	15.4	3.0	8.2
ORF	12.0	15.5	5.0	8.6

The consensus length of the ORF was found to be 7002 nucleotides, including the stop codon. Several amino acid insertions and deletions were observed in various regions of the genome among different FMDV serotype A isolates collected across the globe. A single amino acid insertion at codon position 25 in the L region was identified in three isolates from Kenya (1964), Chad (1973), and Egypt (2006), belonging to the A/Africa/G-17 lineage. Additionally, a two-amino acid insertion at codon positions 30 and 31 in the L region was found in 13 isolates from Turkey (2005–06), Pakistan (2006, 09), Iraq (2009), and Egypt (2012), belonging to the A/ASIA/G-26 (Iran-05) lineage. A three-amino acid deletion at codon positions 25–27 in the L region was observed in 14 isolates from Egypt (2011–16), Nigeria (2013, 2015), Algeria (2017), Sudan (2018), Ethiopia (2019), and Iraq (2022), belonging to the A/AFRICA/G-IV lineage. In the VP3 region, a single amino acid deletion at position 59 was the characteristic signature of all isolates of the G-18/VP3^59^-deletion group from India. In the VP1 region, a deletion at position 140 was found in 43 isolates from diverse countries including Thailand, Vietnam, Malaysia, South Korea, Italy, Germany, France, Spain, Argentina, Russia, and Laos, sampled between 1942 and 2016. Additional deletions at positions 142 and 143 in VP1 were identified in eight and six isolates, respectively, sampled from European and South American countries during the mid-20th century. These VP1 changes are located within the major antigenic βG-βH loop, near the cellular receptor-binding RGD motif. In the 3A region, deletions were noted at position 128 in a single Sudanese isolate (2018), at position 132 in seven isolates from Algeria, Nigeria, and Russia, and at position 142 in two Argentinian isolates from 1959 and 1961. All these positions in 3A are located within the C-terminal third (amino acids 127–151), a region previously identified as highly flexible, hypervariable, and under positive selection (Mohapatra et al. 2011b).

### Genome characteristics of Indian isolates

The length of FMDV serotype A Indian isolates sequenced in this study varied from 8129 to 8179 nt excluding the poly C tract and poly A tail. The variability in the genome length is mainly due to deletion/insertion in the UTRs. Length of the SF-UTR fragment varied between 369 and 371 nucleotides with a consensus length of 370 nucleotides. No chunk deletion was observed in any of the isolates in SF-UTR. In an earlier study, a large stretch of 41 nt deletion in the SF-UTR was reported in one of the VP3^59^-deletion group viruses (Subramaniam et al. 2011). In LF-UTR, a block deletion of nucleotides was observed in three FMDV isolates of the novel lineage. A stretch of 43 nt deletion was observed in two isolates (AIC169/2019 and AIC195/2022), and 16 nt was found deleted in one isolate (AIC370/2021). Similarly, a block deletion of 46 nucleotides was observed in one (IND53/2008) of the nondeletion group isolates sequenced in this study. The deletion in IND53/2008 is located 21 nucleotides downstream of the poly C tract. However, in the case of the isolates of the novel lineage, the deletion is observed at the beginning of LF-UTR. All the LF-UTR deletions correspond to the predicted pseudoknot region. The critical residues and functional motifs in the nonstructural protein-coding regions (Koonin 1991, Grubman et al. 1995, Piccone et al. 1995, Xiang et al. 1998, Guarné et al. 2000, Mason et al. 2003, Birtley et al. 2005) were found to be fully conserved in all the isolates.

The percent nucleotide identity among the isolates of the A/ASIA/G-18/2019 lineage at the ORF level ranged from 92.6% to 93.8%. These isolates exhibited the highest nucleotide identity of 92.0% to 92.4% with an isolate of the G-18 VP3^59^-deletion group sampled in 2007 in India. In contrast, they shared the lowest sequence identity of 83.7%–83.9% with an isolate of A/AFRICA/G-IV (G-15), collected in 2011. Additionally, the isolates from the 2019 novel lineage demonstrated a similar level of sequence identity of 90.9%–91.1% with the currently used serotype A Indian vaccine strain IND40/2000 and 89.2%–89.8% with the proposed candidate vaccine strain IND27/2011. All the isolates of A/ASIA/G-18/2019 lineage exhibited a closed antigenic match with the IND27/2011 strain, whereas most showed antigenic divergence from the vaccine strain in use, IND40/2000 as indicated by r-values from the 2-dimensional virus neutralization test (Table 5) (Mohapatra et al. 2023). In comparison to IND40/2000, all four A/ASIA/G-18/2019 lineage viruses showed changes at 8, 5, and 2 amino acid positions in VP1, VP2, and VP3, respectively. Similarly, compared to IND27/2011, 3, 4, and 6 amino acid positions in VP1, VP2, and VP3, respectively, were found to be altered. Further, the viruses of the new genetic lineage showed 3, 2, and 4 aa changes in VP1, VP2, and VP3, respectively, compared to both the vaccine strains. Out of those aa positions, four in VP1 (83, 139, 154, and 170), one in VP2 (133), and one in VP3 (59) were reported to be antigenically critical. The A/ASIA/G-18/2019 viruses showed variations at four antigenically critical positions, *viz* 83, 154 and 170 of VP1, and 59 of VP3 with reference to vaccine strain IND40/2000. However, when compared to IND27/2011, changes were observed at only two positions, *viz* VP1–139 and VP2–133. It is important to note that predicting antigenic phenotype based solely on capsid sequences remains a paradigmatic challenge, as numerous unidentified antigenic sites likely exist, and even distant amino acid residues may subtly influence epitope structure. The correlation between sequence variation and antigenic properties observed in this study is preliminary and requires validation using monoclonal antibody-resistant mutants and a larger dataset of field isolate sequences.

**Table 5:** Antigenic relationship value of serotype A A/ASIA/G-18/2019 isolates with currently used vaccine strain A/IND40/2000 and candidate vaccine strain A/IND27/2011 (Mohapatra et al. 2023)

S No	Isolates	r1 value with IND40/2000	r1 value with IND27/2011
1	A/IC370/2021	0.51	0.74
2	A/IC161/2021	0.41	0.93
3	A/IC195/2022	0.19	0.70

## Discussion

The study provides a comprehensive, genome-wide phylogenomic and evolutionary analysis of FMDV serotype A on a global scale, incorporating 22 Indian serotype A isolates whose sequences were determined in this study, including 4 from the novel lineage that emerged in 2019 (Mohapatra et al. 2023). The Indian virus sequences are compared with previously published 151 genomic sequences from various parts of the world to deduce the molecular epidemiological and evolutionary trend of serotype A FMDV. The isolates analysed in this study clustered within the three continental topotypes, *viz* including Asia, Africa, and Euro-SA, each having subclusters in the form of region-specific genotypes and lineages as described earlier (Mohapatra et al. 2011a). Key dominating lineages that have left significant epidemiological footprints include A/ASIA/Iran-05 (G-26) in the Middle East and Asia, A/ASIA/G-18 (ASIA/VII) in South Asia, and A/AFRICA/G-IV in Africa. These genetic groups differ by over 15% in VP1 nucleotide sequences and display unique molecular signatures, including amino acid insertions and deletions in the structural and nonstructural proteins. All the Indian isolates sequenced in this study were grouped within genotype 18 (ASIA/VII), with 14 isolates in the VP3^59^-deletion group, 4 isolates in the nondeletion group, and 4 most recent isolates in the novel ‘A/ASIA/G-18/2019’ lineage. At the complete ORF-based maximum likelihood phylogeny, the ‘A/ASIA/G-18/2019’ lineage also maintained its genetic distinctness, akin to VP1 region tree topology, clustering separately from the other two lineages within genotype 18.

The mean rate of evolutionary change for the ORF was 2.369 × 10^−3^ substitutions per site per year (s/s/y). The capsid region's evolutionary rate (3.086 × 10^−3^ s/s/y) aligns closely with that of the ORF, reflecting its rapid evolution. Evolutionary rates varied significantly among the three topotypes. The relatively faster evolutionary rate observed in the Asia topotype possibly reflects strong selective pressures due to disease endemicity; vaccination strategies adopted; and a vast, dense, and diverse population of susceptible animals, resulting in continued emergence and active circulation of new lineages in regions like South Asia, the Middle East, and Southeast Asia. In the case of Africa topotype, the estimated evolutionary rate suggests a slower but sustained evolutionary process. The relatively low evolutionary rate for the topotype EURO-SA is consistent with viral stasis and lack of recent diversification. A similar observation on the differential rate of evolution among the three continental topotypes was also made earlier (Xu and Yang 2021).

The EURO-SA topotype is predicted to be the oldest, with tMRCA around 1873.2 CE, reflecting its diverse genotypes, followed by the Asia topotype with tMRCA estimated at 1914.6 CE, indicating more recent monophyletic diversification, and the Africa topotype with tMRCA around 1914.4 CE, suggesting almost concurrent divergence with the Asia topotype. In our study, the ancestral root state for serotype A, based on ORF analysis, could not be specifically determined, as Argentina and Germany showed nearly equal probability values. An earlier analysis integrating the global VP1 sequences suggested that the common ancestor of serotype A isolates existed around 50 years earlier than estimated in the present study, approximately in the year 1823 (Tully and Fares 2008). The study proposed that the genesis of FMDV serotype A was likely in Europe, and the disease spread to South America through European exploration. The Asia and Africa topotypes evolved from the European root, and the relatively younger tMRCA estimates for Asia and Africa topotypes suggest their active evolution and adaptation to endemic regions. The steady increase in the effective number of infections from the year 1950 to 1995 indicates a period of sustained epidemic growth and viral spread. Around 2000, the rate of spread significantly dropped, suggesting successful interventions including vaccination or ecological factors limiting viral transmission. A sharp but transient increase in relative genetic diversity occurred from 2004 to 2006, likely driven by the emergence of new genetic variants, leading to a temporary expansion of viral diversity. Following the 2004–06 peak, the effective population size declined, indicating purifying selection, where less fit strains were eliminated due to preexisting immunity prevailing in the host population, leading to stabilization of overall genetic diversity.

The majority of codon sites across genetic regions appear to be under negative selection, underscoring the genome’s tendency to preserve essential functions by eliminating deleterious mutations. Episodic selection was identified at 82 codon sites, which suggests sporadic adaptive changes in response to environmental pressures, such as host immune responses or ecological factors (Canário Viana et al. 2022). The identification of antigenically critical sites in the capsid under diversifying selection highlights the role of host immunity in shaping viral diversity, driving adaptation at key functional regions like the ßG-ßH loop of VP1. For single-stranded RNA viruses, recombination is a major evolutionary mechanism through which an isolate can rapidly adapt to new environmental conditions and hosts, thereby widening its ecological niche. Viable recombination events are reported to occur more frequently in the nonstructural protein-coding region and less commonly in the structural protein-coding region (Tosh et al. 2002, Carrillo et al. 2005, Ferretti et al. 2018). Out of 13 recombination events detected, only 5 events showed overlap in time and space. It's important to note that recombination events inferred from sequence analysis should be interpreted cautiously and considered only when supported by circumstantial evidence gathered from virus circulation within a specified spatiotemporal framework. Major and minor parental gene pools represent the possible sources of the majority and minority of genetic material in the recombinant progeny but may not always be the actual progenitors.

Structural protein-coding regions displayed higher genetic variability compared to nonstructural protein-coding regions. The observed sequence conservation in the nonstructural protein coding region barring L and 3A is not unusual and suggests functional and structural constraints on diversity. Insertions and deletions of amino acids were observed in various genomic regions of the serotype A isolates, particularly in the L, VP1, and 3A regions indicating significant evolutionary events occurring in the dispensable parts of the genome, which may confer advantages in genetic diversity and adaptability to the virus. Amino acid insertion and deletion were reported to occur commonly in serotype A isolates (Malirat et al. 2012) without affecting viral fitness. Block deletions in the UTR region were found to disrupt the genomic organization of pseudoknot (PK)-I and PK-II structural elements. However, variations in the number and redundancy of PKs had been documented to have no effect on virus replication, neither in cattle nor in BHK21 cells. Most of the natural isolates were found to maintain at least two PKs even after carrying large deletions in the LF-UTR. The specific roles of these PKs in virus biology have not yet been elucidated in detail. The conservation level of the PK-IV was shown to be high when compared to other PKs located before it in tandem (Mohapatra et al. 2008). The sequences deduced in this study also revealed deletions that presumably affect the structural integrity of pseudoknots without interfering in the tertiary structures of the Internal Ribosome Entry Site (IRES) elements, critical to the viral genome translation.

Nevertheless, the present study has certain limitations that need to be acknowledged. Firstly, the dataset is predominantly Asia-centric, which may restrict the scope of generalization and extrapolation of the findings to a broader global context. The underrepresentation of complete genome sequences from other regions, particularly contemporaneous sequences from Africa and South America, constrains the ability to draw comprehensive conclusions about the global evolutionary dynamics and for mapping transboundary transmission patterns of FMDV serotype A. In addition, incomplete or missing metadata, such as precise geographic locations, host details, and exact collection dates for several isolates, hinder high-resolution epidemiological and phylogeographic interpretations. These deficiencies highlight the need for geographically diverse sampling, improved metadata collection, and real-time availability of complete genome sequences to derive more precise evolutionary insights and to better understand the global diversity and transmission corridors of FMDV.

## Conclusions

In this study, complete genome sequences of 22 serotype A Indian FMD viruses were generated and analysed alongside 151 serotype A sequences retrieved from GenBank. Evolutionary rates varied significantly among the three topotypes, with the Asia topotype showing a higher rate, probably due to active widespread circulation of the virus and the ongoing emergence of new lineages. Frequent amino acid insertions and deletions were found in the L, VP1, and 3A regions of serotype A isolates without affecting viral fitness. Inter- and intragenotypic recombination events were identified in 13 isolates, suggesting it to be one of the important mechanisms of virus evolution. At the complete ORF based maximum likelihood phylogeny, the novel ‘A/ASIA/G-18/2019’ lineage that emerged in India during 2019 also maintained its genetic distinctness as observed in VP1 tree topology, clustering separately from the other two lineages within genotype 18. The established functionally critical motifs were found to be conserved in the novel lineage. Except for a block deletion in the LF-UTR, presumably in the pseudoknot region, the genome of the novel lineage revealed no deletions or insertions in the coding region. These insights enhance our understanding of the evolutionary dynamics of FMDV serotype A virus in a global context.

## Supplementary Material

Supplementary_Figure_S4_veaf097

Supplementary_Table_S1_veaf097

Supplementary_Table_S2_veaf097

Supplementary_Table_S3_veaf097

## Data Availability

All required data are available as texts and figures in the main text of the article or in the Supplementary Materials. The sequence data sets generated during this research are publicly available at NCBI GenBank.

## References

[ref1] Birtley JR, Knox SR, Jaulent AM et al. Crystal structure of foot-and-mouth disease virus 3C protease. New insights into catalytic mechanism and cleavage specificity. *J Biol Chem* 2005;280:11520–7. 10.1074/jbc.M413254200 15654079

[ref2] Canário Viana MV, Profeta R, Cerqueira JC et al. Evidence of episodic positive selection in Corynebacterium diphtheriae complex of species and its implementations in identification of drug and vaccine targets. *PeerJ* 2022;10:e12662. 10.7717/peerj.12662 35190783 PMC8857904

[ref3] Carrillo C, Tulman ER, Delhon G et al. Comparative genomics of foot-and-mouth disease virus. *J Virol* 2005;79:6487–504. 10.1128/JVI.79.10.6487-6504.2005 15858032 PMC1091679

[ref4] Ferretti L, Di Nardo A, Singer B et al. Within-host recombination in the foot-and-mouth disease virus genome. *Viruses* 2018;10:221. 10.3390/v10050221 29693634 PMC5977214

[ref5] Grubman MJ, Zellner M, Bablanian G et al. Identification of the active-site residues of the 3C proteinase of foot-and-mouth disease virus. *Virology* 1995;213:581–9. 10.1006/viro.1995.0030 7491782

[ref6] Guarné A, Hampoelz B, Glaser W et al. Structural and biochemical features distinguish the foot-and-mouth disease virus leader proteinase from other papain-like enzymes. *J Mol Biol* 2000;302:1227–40. 10.1006/jmbi.2000.4115 11183785

[ref7] Hagag NM, Hassan AM, Zaher MR et al. Molecular detection and phylogenetic analysis of newly emerging foot-and-mouth disease virus type a, lineage EURO-SA in Egypt in 2022. *Virus Res* 2023;323:198960. 10.1016/j.virusres.2022.198960 36209919 PMC10194312

[ref8] Jangra RK, Tosh C, Sanyal A et al. Antigenic and genetic analyses of foot-and-mouth disease virus type a isolates for selection of candidate vaccine strain reveals emergence of a variant virus that is responsible for most recent outbreaks in India. *Virus Res* 2005;112:52–9. 10.1016/j.virusres.2005.03.021 16022900

[ref9] Kalyaanamoorthy S, Minh BQ, Wong TKF et al. ModelFinder: Fast model selection for accurate phylogenetic estimates. *Nat Methods* 2017;14:587–9. 10.1038/nmeth.4285 28481363 PMC5453245

[ref10] Katoh K, Rozewicki J, Yamada KD. MAFFT online service: Multiple sequence alignment, interactive sequence choice and visualization. *Brief Bioinform* 2019;20:1160–6. 10.1093/bib/bbx108 28968734 PMC6781576

[ref11] Koonin EV . The phylogeny of RNA-dependent RNA polymerases of positive-strand RNA viruses. *J Gen Virol* 1991;72:2197–206. 10.1099/0022-1317-72-9-2197 1895057

[ref12] Lemey P, Rambaut A, Drummond AJ et al. Bayesian phylogeography finds its roots. *PLoS Comput Biol* 2009;5:e1000520. 10.1371/journal.pcbi.1000520 19779555 PMC2740835

[ref13] Malirat V, Bergmann IE, de Mendonça CR et al. Molecular epidemiology of foot-and-mouth disease virus type a in South America. *Vet Microbiol* 2012;158:82–94. 10.1016/j.vetmic.2012.02.009 22397938

[ref14] Martin DP, Varsani A, Roumagnac P et al. RDP5: A computer program for analyzing recombination in, and removing signals of recombination from, nucleotide sequence datasets. *Virus Evol* 2020;7:veaa087. 10.1093/ve/veaa087 33936774 PMC8062008

[ref15] Mason PW, Pacheco JM, Zhao QZ et al. Comparisons of the complete genomes of Asian, African and European isolates of a recent foot-and-mouth disease virus type O pandemic strain (PanAsia). *J Gen Virol* 2003;84:1583–93. 10.1099/vir.0.18669-0 12771429

[ref16] Mohapatra JK, Sanyal A, Hemadri D et al. Comparative genomics of serotype Asia 1 foot-and-mouth disease virus isolates from India sampled over the last two decades. *Virus Res* 2008;136:16–29. 10.1016/j.virusres.2008.04.010 18511143

[ref17] Mohapatra JK, Subramaniam S, Pandey LK et al. Phylogenetic structure of serotype a foot-and-mouth disease virus: Global diversity and the Indian perspective. *J Gen Virol* 2011a;92:873–9. 10.1099/vir.0.028555-0 21228130

[ref18] Mohapatra JK, Pawar SS, Tosh C et al. Genetic characterization of vaccine and field strains of serotype a foot-and-mouth disease virus from India. *Acta Virol* 2011b;55:349–52. 10.4149/av_2011_04_349 22149500

[ref20] Mohapatra JK, Dahiya SS, Subramaniam S et al. Emergence of a novel genetic lineage 'a/ASIA/G-18/2019′ of foot and mouth disease virus serotype a in India: A challenge to reckon with. *Virus Res* 2023;333:199140. 10.1016/j.virusres.2023.199140 37268276 PMC10352718

[ref21] Piccone ME, Zellner M, Kumosinski TF et al. Identification of the active-site residues of the L proteinase of foot-and-mouth disease virus. *J Virol* 1995;69:4950–6. 10.1128/jvi.69.8.4950-4956.1995 7609064 PMC189310

[ref22] Rambaut A, Lam TT, Max Carvalho L et al. Exploring the temporal structure of heterochronous sequences using TempEst (formerly path-O-gen). *Virus Evol* 2016;2:vew007. 10.1093/ve/vew007 27774300 PMC4989882

[ref23] Subramaniam S, Sanyal A, Mohapatra JK et al. Comparative complete genome analysis of Indian type a foot-and-mouth disease virus field isolates. *Virus Genes* 2011;43:224–33. 10.1007/s11262-011-0622-8 21604149

[ref24] Subramaniam S, Mohapatra JK, Sahoo NR et al. Foot-and-mouth disease status in India during the second decade of the twenty-first century (2011-2020). *Vet Res Commun* 2022;46:1011–22. 10.1007/s11259-022-10010-z 36190601 PMC9527732

[ref25] Suchard MA, Lemey P, Baele G et al. Bayesian phylogenetic and phylodynamic data integration using BEAST 1.10. *Virus Evol* 2018;4:vey016. 10.1093/ve/vey016 29942656 PMC6007674

[ref26] Tamura K, Stecher G, Kumar S. MEGA11: Molecular evolutionary genetics analysis version 11. *Mol Biol Evol* 2021;38:3022–7. 10.1093/molbev/msab120 33892491 PMC8233496

[ref27] Tosh C, Hemadri D, Sanyal A. Evidence of recombination in the capsid-coding region of type a foot-and-mouth disease virus. *J Gen Virol* 2002;83:2455–60. 10.1099/0022-1317-83-10-2455 12237427

[ref28] Tully DC, Fares MA. The tale of a modern animal plague: Tracing the evolutionary history and determining the time-scale for foot and mouth disease virus. *Virology* 2008;382:250–6. 10.1016/j.virol.2008.09.011 18945462

[ref29] Weaver S, Shank SD, Spielman SJ et al. Datamonkey 2.0: A modern web application for characterizing selective and other evolutionary processes. *Mol Biol Evol* 2018;35:773–7. 10.1093/molbev/msx335 29301006 PMC5850112

[ref30] Xiang W, Cuconati A, Hope D et al. Complete protein linkage map of poliovirus P3 proteins: Interaction of polymerase 3Dpol with VPg and with genetic variants of 3AB. *J Virol* 1998;72:6732–41. 10.1128/JVI.72.8.6732-6741.1998 9658121 PMC109881

[ref31] Xu W, Yang M. Genetic variation and evolution of foot-and-mouth disease virus serotype a in relation to vaccine matching. *Vaccine* 2021;39:1420–7. 10.1016/j.vaccine.2021.01.042 33526282

