# Supplementary material for: Full genome–based evolutionary analyses of FMD virus serotype A including field outbreak strains isolated from India during the period 2008–22
Source: Virus Evol. 2025 Dec 18;12(1):veaf097. doi: 10.1093/ve/veaf097 (PMC12821355; doi:10.1093/ve/veaf097)
Supplement: Supplementary_Figure_S4_veaf097 [file supplementary_figure_s4_veaf097.pptx]

## Slide 1
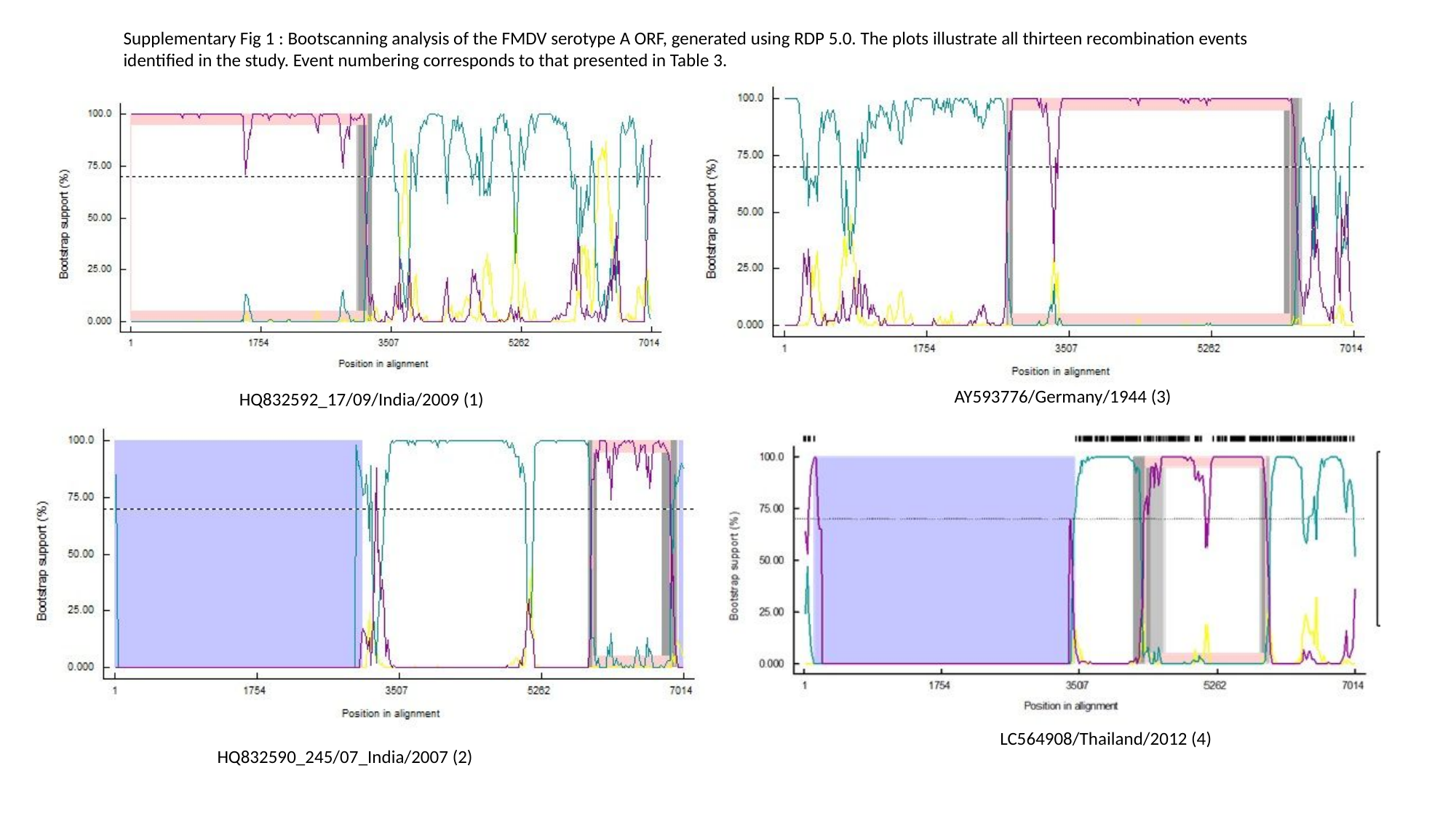

Supplementary Fig 1 : Bootscanning analysis of the FMDV serotype A ORF, generated using RDP 5.0. The plots illustrate all thirteen recombination events identified in the study. Event numbering corresponds to that presented in Table 3.
AY593776/Germany/1944 (3)
HQ832592_17/09/India/2009 (1)
LC564908/Thailand/2012 (4)
HQ832590_245/07_India/2007 (2)

## Slide 2
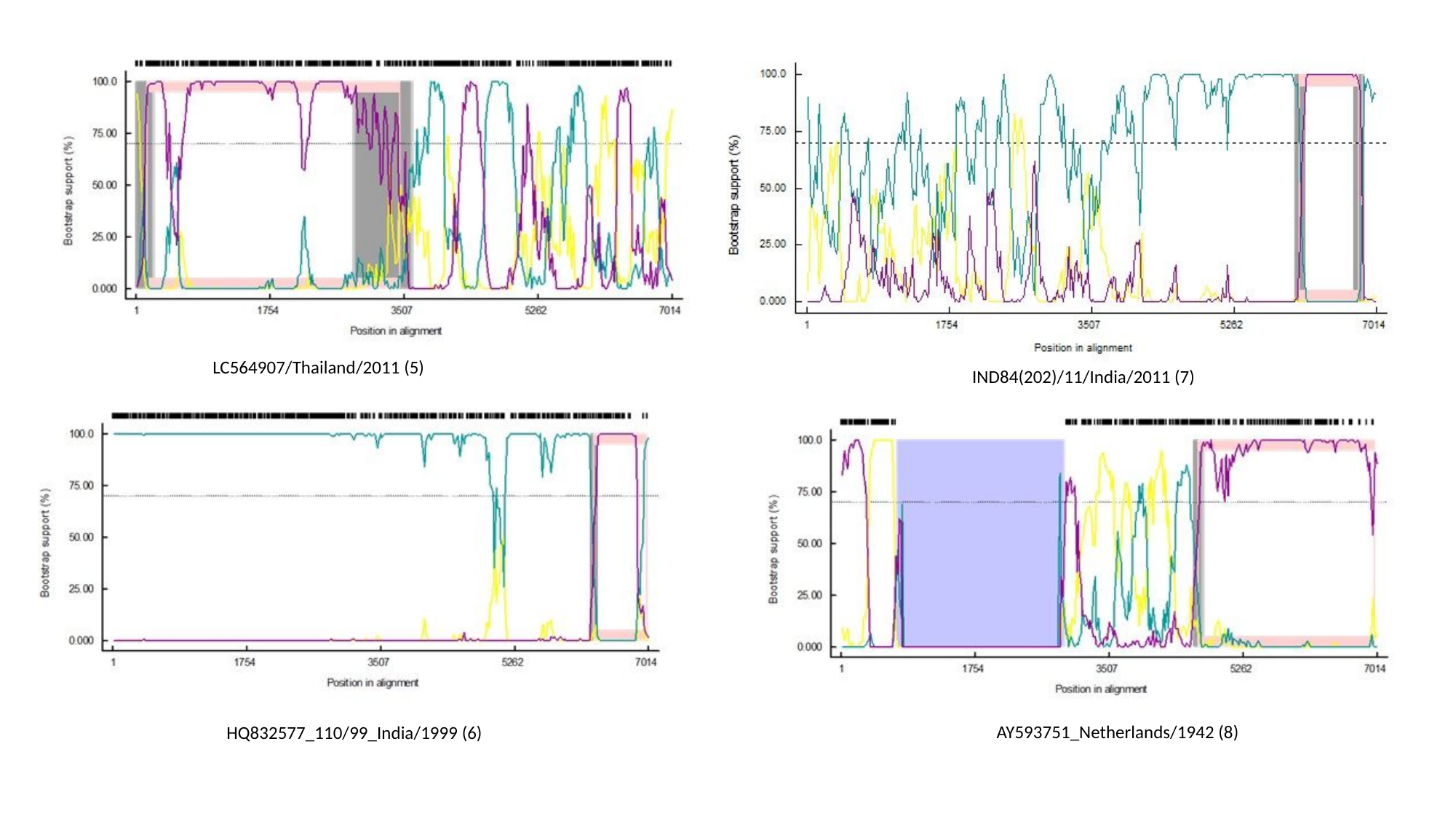

LC564907/Thailand/2011 (5)
IND84(202)/11/India/2011 (7)
AY593751_Netherlands/1942 (8)
HQ832577_110/99_India/1999 (6)

## Slide 3
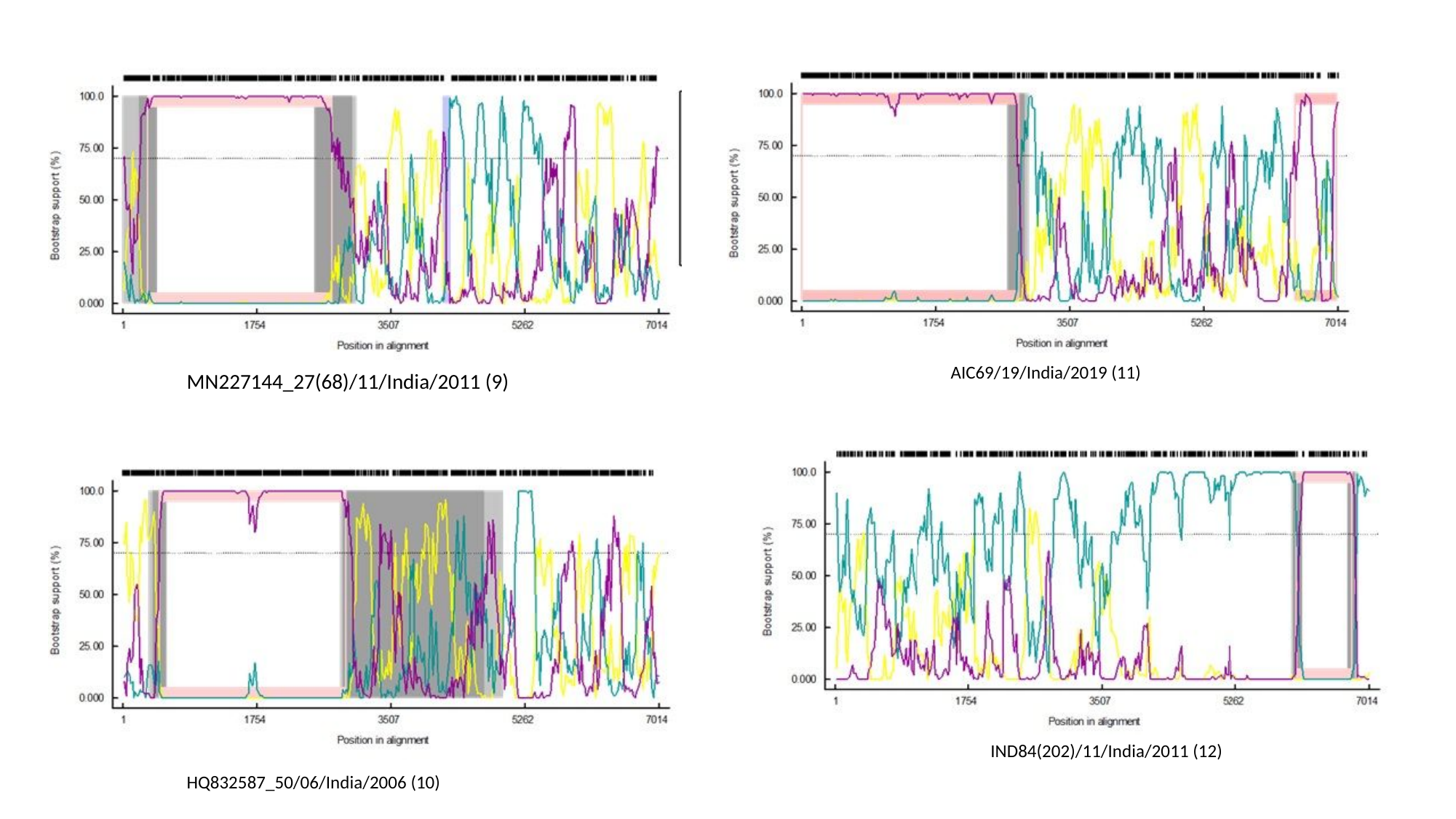

AIC69/19/India/2019 (11)
MN227144_27(68)/11/India/2011 (9)
IND84(202)/11/India/2011 (12)
HQ832587_50/06/India/2006 (10)

## Slide 4
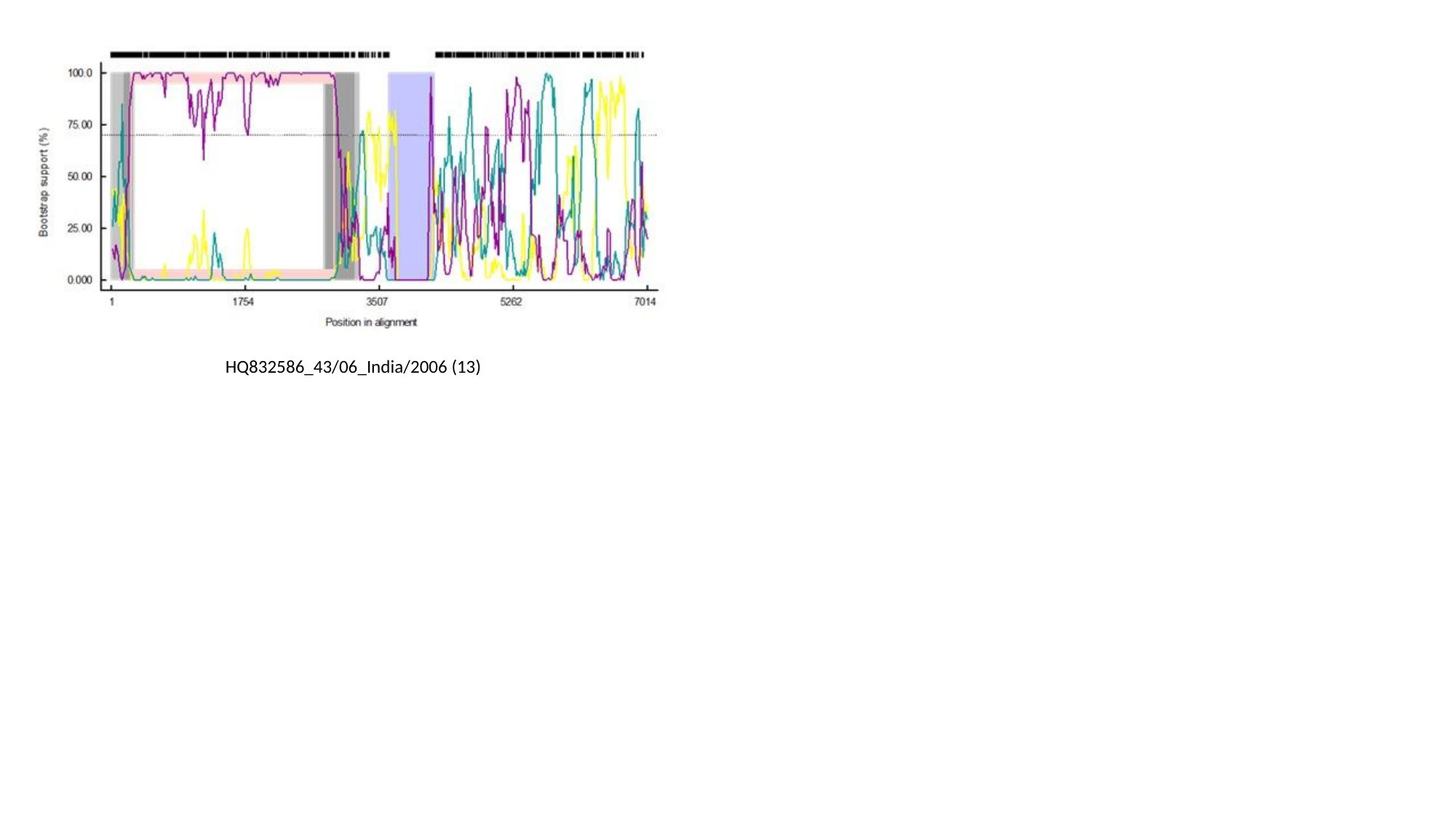

HQ832586_43/06_India/2006 (13)
