# Supplementary material for: Full genome–based evolutionary analyses of FMD virus serotype A including field outbreak strains isolated from India during the period 2008–22
Source: Virus Evol. 2025 Dec 18;12(1):veaf097. doi: 10.1093/ve/veaf097 (PMC12821355; doi:10.1093/ve/veaf097)
Supplement: Supplementary_Table_S1_veaf097 [file supplementary_table_s1_veaf097.docx]

**Table S1.** History of serotype A isolates sequence determined in this study

|  | Isolate ID | Place of Outbreak | Species | Date of Collection | Accession Number |
| --- | --- | --- | --- | --- | --- |
| 1 | IND61/1988 | Kolkata, West Bengal | Elephant | 1988 | PQ768001 |
| 2 | IND53/2008 | Raipur, Chhattisgarh | Cattle | 14-Jan-08 | PQ768002 |
| 3 | IND165/2008 | Agroha, Haryana | Cattle | 24-March-08 | PQ768003 |
| 4 | IND436/2008 | Kolar, Karnataka | Cattle | 16-May-08 | PQ768004 |
| 5 | IND11/2009 | Kollam, Kerala | Cattle | 2009 | PQ768005 |
| 6 | IND832/2009 | Ludhiana, Punjab | Cattle | 13-Dec-09 | PQ768006 |
| 7 | IND1(3)/2010 | Kashipur, Uttarakhand | Cattle | 03-Jan-10 | PQ768007 |
| 8 | IND46(98)/2010 | Kurukshetra, Haryana | Cattle | 27-Jan-10 | PQ768008 |
| 9 | IND226(616)/2010 | Guntur, Andhra Pradesh | Cattle | 16-Oct-10 | PQ768009 |
| 10 | IND84(202)/2011 | Jaipur, Rajasthan | Cattle | 02-Jun-11 | PQ768010 |
| 11 | IND113(213)/2012 | Ahmedabad, Gujarat | Cattle | 2012 | PQ768011 |
| 12 | IND168(388)/2012 | Uttar Pradesh | Buffalo | 09-Apr-12 | PQ768012 |
| 13 | IND264(605)/2012 | Jagatpur, Odisha | Cattle | 08-Jul-12 | PQ768013 |
| 14 | IND270(616)/2012 | Bengaluru Urban, Karnataka | Cattle | 29-Jun-12 | PQ768014 |
| 15 | IND307(663)/2012 | Kamrup, Assam | Cattle | 16-Jul-12 | PQ768015 |
| 16 | IND404(828)/2012 | Ramanagara, Karnataka | Cattle | 14-Sep-12 | PQ768016 |
| 17 | IND107(199)/2013 | Ranchi, Jharkhand | Cattle | 23-Feb-13 | PQ768017 |
| 18 | IND153(332)/2013 | Netravali, Goa | Cattle | 07-Feb-13 | PQ768018 |
| 19 | A/IC69/2019 | Satara, Maharashtra | Cattle | Jan-2019 | PQ768019 |
| 20 | A/IC370/21 | Brajrajnagar, Odisha | Cattle | 12-Aug-21 | PQ768020 |
| 21 | A/IC161/21 | Kupwara, Jammu & Kashmir | Cattle | 09-Jun-21 | PQ768021 |
| 22 | A/IC195/22 | Jajpur, Odisha | Cattle | 23-Feb-22 | PQ768022 |
