# Supplementary material for: Full genome–based evolutionary analyses of FMD virus serotype A including field outbreak strains isolated from India during the period 2008–22
Source: Virus Evol. 2025 Dec 18;12(1):veaf097. doi: 10.1093/ve/veaf097 (PMC12821355; doi:10.1093/ve/veaf097)
Supplement: Supplementary_Table_S2_veaf097 [file supplementary_table_s2_veaf097.docx]

**Table S2:** Details of FMDV serotype A isolates used in this study

| **S No** | **Accession Number** | **Country** | **Year of collection** |
| --- | --- | --- | --- |
|  | AY593752 | United Kingdom | 1932 |
|  | AY593751 | Netherlands | 1942 |
|  | AY593759 | Germany | 1942 |
|  | AY593774 | Spain | 1943 |
|  | AY593776 | Germany | 1944 |
|  | AY593779 | Germany | 1944 |
|  | AY593777 | Germany | 1948 |
|  | AY593792 | Italy | 1950 |
|  | AY593781 | Germany | 1951 |
|  | AY593768 | Brazil | 1955 |
|  | AY593784 | Argentina | 1955 |
|  | AY593753 | Brazil | 1958 |
|  | AY593754 | Spain | 1959 |
|  | AY593756 | Brazil | 1959 |
|  | AY593757 | Brazil | 1959 |
|  | AY593769 | Argentina | 1959 |
|  | AY593778 | Spain | 1959 |
|  | AY593755 | Thailand | 1960 |
|  | AY593760 | Russia | 1960 |
|  | AY593780 | France | 1960 |
|  | AY593789 | Argentina | 1961 |
|  | AY593762 | Iraq | 1964 |
|  | AY593763 | Iraq | 1964 |
|  | AY593764 | Iraq | 1964 |
|  | AY593761 | Kenya | 1964 |
|  | AY593785 | Argentina | 1964 |
|  | AY593765 | Turkey | 1965 |
|  | X74812 | Azerbaijan | 1965 |
|  | AY593766 | Kenya | 1965 |
|  | AY593767 | Argentina | 1965 |
|  | AY593770 | Argentina | 1966 |
|  | AY593758 | Venezuela | 1967 |
|  | AY593771 | Colombia | 1967 |
|  | AY593772 | Turkey | 1968 |
|  | AY593773 | Peru | 1969 |
|  | AY593775 | Venezuela | 1970 |
|  | MH053305 | Egypt | 1972 |
|  | MH053306 | Chad | 1973 |
|  | AY593793 | Philippines | 1975 |
|  | AY593803 | Brazil | 1976 |
|  | HM854022 | India | 1977 |
|  | AY593788 | Brazil | 1979 |
|  | HM854024 | India | 1982 |
|  | AY593794 | Colombia | 1985 |
|  | HQ832576 | India | 1990 |
|  | MH053307 | Zambia | 1990 |
|  | KJ933864 | Malaysia | 1997 |
|  | AY593791 | Iran | 1998 |
|  | FJ623456 | Kazakhstan | 1999 |
|  | HM854023 | India | 1999 |
|  | HQ832577 | India | 1999 |
|  | HM854021 | India | 2000 |
|  | HM854025 | India | 2000 |
|  | AY593782 | Argentina | 2000 |
|  | AY593783 | Argentina | 2001 |
|  | AY593786 | Argentina | 2001 |
|  | AY593790 | Argentina | 2001 |
|  | AY593801 | Uruguay | 2001 |
|  | AY593802 | Uruguay | 2001 |
|  | KY404934 | Argentina | 2001 |
|  | KY404935 | Argentina | 2001 |
|  | MK341544 | Argentina | 2001 |
|  | MK341545 | Argentina | 2001 |
|  | HQ832578 | India | 2003 |
|  | HQ832579 | India | 2003 |
|  | HQ832580 | India | 2003 |
|  | JF749848 | Turkey | 2003 |
|  | HQ268509 | VietNam | 2004 |
|  | HQ832581 | India | 2004 |
|  | HQ832582 | India | 2004 |
|  | EF494486 | Turkey | 2005 |
|  | HQ832583 | India | 2005 |
|  | KY446902 | Pakistan | 2005 |
|  | EF494487 | Pakistan | 2006 |
|  | EF494488 | Pakistan | 2006 |
|  | HQ832584 | India | 2006 |
|  | HQ832585 | India | 2006 |
|  | HQ832586 | India | 2006 |
|  | HQ832588 | India | 2006 |
|  | HQ832589 | India | 2006 |
|  | JF749841 | Turkey | 2006 |
|  | JF749843 | Egypt | 2006 |
|  | HQ632773 | Malaysia | 2007 |
|  | HQ832590 | India | 2007 |
|  | LC483874 | Thailand | 2007 |
|  | HQ832591 | India | 2008 |
|  | JN006722 | Pakistan | 2008 |
|  | GQ406247 | VietNam | 2009 |
|  | GQ406248 | VietNam | 2009 |
|  | GQ406250 | VietNam | 2009 |
|  | GQ406251 | VietNam | 2009 |
|  | HQ832592 | India | 2009 |
|  | JN099688 | Iraq | 2009 |
|  | JN099694 | Iraq | 2009 |
|  | JN099695 | Iraq | 2009 |
|  | JN099697 | Iraq | 2009 |
|  | JN099698 | Iraq | 2009 |
|  | JN099699 | Iraq | 2009 |
|  | LC564901 | Thailand | 2009 |
|  | MN062586 | Afghanistan | 2009 |
|  | MN062587 | Pakistan | 2009 |
|  | KC588943 | SouthKorea | 2010 |
|  | LC564904 | Thailand | 2010 |
|  | LC564907 | Thailand | 2011 |
|  | MN227144 | India | 2011 |
|  | KC440881 | Egypt | 2011 |
|  | KC440882 | Egypt | 2012 |
|  | LC564908 | Thailand | 2012 |
|  | OM863580 | Egypt | 2012 |
|  | OM863581 | Kenya | 2012 |
|  | KJ608371 | VietNam | 2013 |
|  | KJ754939 | Bangladesh | 2013 |
|  | KM268896 | Turkey | 2013 |
|  | KT968663 | China | 2013 |
|  | KY322676 | Malaysia | 2013 |
|  | KY322677 | Malaysia | 2013 |
|  | KY322678 | Malaysia | 2013 |
|  | KY322680 | VietNam | 2013 |
|  | LC564911 | Thailand | 2013 |
|  | LC564912 | Thailand | 2013 |
|  | MG725872 | Nigeria | 2013 |
|  | KY322675 | Laos | 2014 |
|  | KY322679 | Thailand | 2014 |
|  | LC564914 | Thailand | 2014 |
|  | LC564915 | Thailand | 2014 |
|  | LC564916 | Thailand | 2014 |
|  | KP940474 | Egypt | 2014 |
|  | KU127247 | SaudiArabia | 2015 |
|  | LC564917 | Thailand | 2015 |
|  | LC564918 | Thailand | 2015 |
|  | LC564919 | Thailand | 2015 |
|  | LC564920 | Thailand | 2015 |
|  | MG725873 | Nigeria | 2015 |
|  | MG725875 | Nigeria | 2015 |
|  | MG725876 | Nigeria | 2015 |
|  | LC564921 | Thailand | 2016 |
|  | LC564922 | Thailand | 2016 |
|  | LC564923 | Thailand | 2016 |
|  | MK088171 | Bangladesh | 2016 |
|  | OQ745961 | Thailand | 2016 |
|  | MN116688 | Kenya | 2016 |
|  | MT863268 | Egypt | 2016 |
|  | MK940253 | SaudiArabia | 2017 |
|  | MG913340 | Algeria | 2017 |
|  | MG923579 | Algeria | 2017 |
|  | MG923580 | Algeria | 2017 |
|  | MT602077 | Ethiopia | 2018 |
|  | MT602079 | Sudan | 2018 |
|  | MT602078 | Ethiopia | 2019 |
|  | MT602080 | Uganda | 2019 |
|  | LC771233 | Iraq | 2022 |
