# Supplementary material for: Full genome–based evolutionary analyses of FMD virus serotype A including field outbreak strains isolated from India during the period 2008–22
Source: Virus Evol. 2025 Dec 18;12(1):veaf097. doi: 10.1093/ve/veaf097 (PMC12821355; doi:10.1093/ve/veaf097)
Supplement: Supplementary_Table_S3_veaf097 [file supplementary_table_s3_veaf097.docx]

**Table S3.** Summary of genetic groups, country and year (s) of isolation of FMDV serotype A strains (n=173) included in this study

| **Genetic Group** | **Number of isolates** | **Countries** | **Year range of collection** |
| --- | --- | --- | --- |
| **Asia topotype (108)** | | | |
| A/ASIA/G-8 | 1 | Thailand | 1960 |
| A/ASIA/G-10 | 8 | India, Turkey, Iraq, Azerbaijan, Kazakhstan | 1964-1999 |
| A/ASIA/G-16 | 3 | India | 1982-1999 |
| A/ASIA/G-18 | 42 | India, Bangladesh, Saudi Arabia | 2000-2022 |
| A/ASIA/G-20 (Sea-97) | 34 | Malaysia, Thailand, Vietnam, Laos, South Korea, China | 1997-2016 |
| A/ASIA/G-22 (Iran-96) | 2 | Iran, Turkey | 1998-2003 |
| A/ASIA/G-26 (Iran-05) | 15 | Turkey, Pakistan, Afghanistan, Egypt, Iraq | 2006-2013 |
| **EURO-SA topotype (41)** | | | |
| A/EURO-SA/G-2 | 15 | Colombia, Germany, France, Italy, Spain, UK, Netherlands, Argentina, Russia | 1932-1967 |
| A/EURO-SA/G-3 | 3 | Brazil, Colombia | 1955-1985 |
| A/EURO-SA/G-5 | 3 | Brazil, Venezuela | 1958-1967 |
| A/EUSO/SA/G-6 | 2 | Brazil | 1976-1979 |
| A/EURO-SA/G-7 | 2 | Argentina | 1959-1961 |
| A/EUSO/SA/G-12 | 1 | Argentina | 1966 |
| A/EURO-SA/G-13 | 12 | Argentina, Uruguay | 1964-2001 |
| A/EUSO/SA/G-14 | 3 | Venezuela, Peru, Philippines | 1969-1975 |
| **Africa topotype (24)** | | | |
| A/AFRICA/G-IV (G-15) | 14 | Algeria, Nigeria, Sudan, Iraq, Egypt, Ethiopia | 2011-2022 |
| A/AFRICA/G-I | 4 | Uganda, Kenya, Ethiopia, | 2012-2019 |
| A/AFRICA/G-VII(G-17) | 1 | Egypt | 2006 |
| A/AFRICA/G-VIII(G-11) | 1 | Kenya | 1965 |
| A/AFRICA/G-III(G-9) | 1 | Kenya | 1964 |
| A/AFRICA/G-II(G-9) | 1 | Egypt | 1972 |
| A/AFRICA/ND | 1 | Chad | 1973 |
| A/AFRICA/ND | 1 | Zambia | 1990 |
